# The gut–heart axis in coronary artery disease: a scoping and narrative review of sex-based microbial and metabolic disparities

**DOI:** 10.1186/s13293-026-00824-w

**Published:** 2026-01-30

**Authors:** Caroline Chong-Nguyen, Rubén Fuentes Artiles, Thomas Pilgrim, Bahtiyar Yilmaz, Yvonne Döring

**Affiliations:** 1 Department of Cardiology, Bern University Hospital, Inselspital, Freiburgstrasse 20, 3010 Bern, Switzerland; 2 Department of Cardiology, Biel/Bienne Hospital, Biel, Switzerland; 3 Department for BioMedical Research (DBMR), Bern University Hospital, University of Bern, Bern, Switzerland; 4 Department of Visceral Surgery and Medicine, Bern University Hospital, University of Bern, 3010 Bern, Switzerland; 5 Maurice Müller Laboratories, Department for Biomedical Research, University of Bern, 3008 Bern, Switzerland; 6 DZHK (German Center for Cardiovascular Research), Partner Site Munich Heart Alliance, Munich, Germany; 7 Swiss Cardiovascular Center, Division of Angiology, Inselspital, Bern University Hospital, University of Bern, Bern, Switzerland; 8 Institute for Cardiovascular Prevention (IPEK), Ludwig-Maximilians-Universität, Munich, Germany

**Keywords:** Gut microbiota, Metabolites, Sex difference, Coronary artery disease, Trimethyl-amine-N-oxide, Indoxyl sulfate

## Abstract

**Background:**

The gut microbiota significantly influences cardiovascular health by regulating host metabolism and generating bioactive compounds like trimethylamine-N-oxide (TMAO) and indoxyl sulfate (IS), both linked to coronary artery disease (CAD). Emerging research indicates sex-based differences in microbial composition and metabolite production, yet their impact on CAD pathophysiology remains unclear. This scoping review summarizes current findings on sex-specific microbial and metabolic differences in individuals with CAD.

**Methods:**

A systematic search of PubMed and EMBASE was conducted through March 2025 for peer-reviewed studies comparing gut microbiota or metabolite profiles between male and female patients with CAD. Eligible studies used 16S rRNA sequencing, shotgun metagenomics, or metabolite profiling to analyze microbial communities and atherosclerosis-associated metabolites. Mechanistic links from genetics, epigenetics, and hormone–microbiota interactions were integrated to provide a more comprehensive understanding of how gut microbiota may contribute to sex differences in CAD.

**Results:**

Eleven studies met the inclusion criteria for this review. Men with CAD exhibited increased relative abundances of taxa such as *Prevotella*, *Clostridia*_UCG_014, UCG_010, and other pro-inflammatory genera, whereas women microbiota was comparatively enriched in *Barnesiella, Bifidobacteriales*, and other potentially beneficial taxa. Parallel differences emerged in microbial metabolite profiles: men demonstrated elevated plasma levels of TMAO and IS, both associated with heightened cardiovascular risk and disease burden. Conversely, women with CAD had higher circulating levels of secondary bile acids and lower TMAO concentrations.

**Conclusion:**

Preliminary studies suggest sex-related differences in gut microbiota composition and metabolite profiles in CAD patients. Integrating mechanistic links from microbial metabolism, genetics, epigenetics, and hormones supports a potential role of the microbiota in sex-dependent disease pathways. Current evidence is limited and mostly observational; well-designed studies are needed to clarify mechanisms, clinical relevance of sex-specific microbiome signatures and specifically assess whether these sex-specific microbial and metabolic differences influence CAD progression and outcomes.

**Supplementary Information:**

The online version contains supplementary material available at 10.1186/s13293-026-00824-w.

## Highlights

- Male and female patients with CAD exhibit distinct gut microbiota compositions, with men showing higher abundances of *Clostridia* genera and *Prevotella* and women enriched in *Barnesiella* and *Bifidobacteriales*.
- Sex-specific differences were also observed in gut-derived metabolites: men had higher levels of TMAO and indoxyl sulfate, while women exhibited increased secondary bile acids and lower TMAO.
- TMAO levels are elevated in women during acute myocardial infarction (AMI) but tend to be higher in men with stable CAD, particularly with advancing age.

**Supplementary Information:**

The online version contains supplementary material available at 10.1186/s13293-026-00824-w.

## Plain Language Summary

Men and women develop heart disease differently, and one possible explanation may lie in the gut microbiome—the community of bacteria living in our digestive system. These bacteria help digest food, regulate metabolism, and influence inflammation, all of which can affect the health of the heart’s arteries. Research on how the gut microbiome differs between men and women with coronary artery disease is still very limited, but early evidence suggests that men and women may have different gut bacterial communities. Men tend to have more bacteria linked to inflammation, while women often have bacteria that may support a healthier gut and immune system. These differences could influence how the body responds to inflammation, processes fats, and maintains healthy blood vessels over time. Although this is an emerging field and we are only beginning to understand these patterns, the findings suggest that the gut microbiome may play a role in why heart disease develops differently in men and women. More research is needed to confirm these observations and to explore whether understanding gut bacteria could lead to new, sex-specific approaches to prevent or treat heart disease in the future.

**Supplementary Information:**

The online version contains supplementary material available at 10.1186/s13293-026-00824-w.

## Introduction

The human microbiome consists of trillions of microorganisms, primarily residing in the gastrointestinal (GI) tract, where they perform critical functions such as fermenting indigestible fibers, regulating immune responses, synthesizing vitamins, aiding energy metabolism, and maintaining gut barrier integrity [1]. Recent metagenomic sequencing has identified over 2,000 species within the gut microbiome, with *Bacillota* and *Bacteroidota* representing over 90% of the population [2]. Dysbiosis, or chronic microbial imbalance, has been linked to various health issues, including cardiovascular disease (CVD) [3].

Beyond compositional changes, gut microbiota exerts its systemic influence through the production of bioactive metabolites. Among these, short-chain fatty acids (SCFAs), trimethylamine-N-oxide (TMAO), and indoxyl sulfate (IS) have emerged as key mediators of host-microbiota interactions relevant to cardiometabolic health [4]. TMAO is generated from dietary choline, carnitine, and betaine via microbial conversion to trimethylamine (TMA), followed by hepatic oxidation by flavin-containing monooxygenases (FMO3) [5]. Elevated plasma TMAO levels have been robustly associated with atherosclerosis, myocardial infarction, and stroke [6–8]. Similarly, IS, a uremic toxin derived from microbial tryptophan metabolism, promotes vascular inflammation, endothelial dysfunction, and plaque instability [9]. Notably, changes in the *Bacillota* to *Bacteroidota* ratio, along with imbalances in these metabolites, are strongly associated with CVD pathogenesis [10].

Sex hormones influence both microbiota composition and host metabolism and may modulate the generation and impact of these microbial metabolites. Yet, the role of sex-specific microbial and metabolic profiles in coronary artery disease (CAD) remains poorly defined. Sex differences in the human intestinal microbiome may explain discrepancies in traditional atherosclerosis risk factors like diabetes, hypertension, dyslipidemia, and obesity. For instance, studies in rats have shown that sex hormones like estradiol modulate the microbiome, with menopause increasing CVD risk through changes in gut microbiota composition. Sex differences in the microbiome have been observed, with certain microbial taxa varying between men and women [11]. These differences influence metabolic profiles, such as lipid levels and body mass index (BMI), with some studies suggesting a greater impact of microbiota on these factors in men. Additionally, sex hormones play a significant role in shaping the microbiome, with alterations in hormone levels resulting in long-lasting changes in microbial diversity, such as shifts in the *Bacillota* to *Bacteroidota* ratio [12, 13].

Understanding these differences in microbiome composition is essential for explaining sex-based discrepancies in diseases like atherosclerosis. Sex hormones influence both microbiome composition and its metabolic activity, which, in turn, affects disease processes [14, 15]. This scoping review examines how sex-specific gut microbial and metabolic profiles may influence CAD risk, progression, and outcomes.

## Methods

### Study protocol and registration

This scoping review adhered to the criteria of the Preferred Reporting Items for Scoping Reviews and Meta-Analyses extension for scoping reviews (PRISMA-ScR) [16], and the protocol was registered with the Open Science Foundation [17]. We conducted a scoping review following the PRISMA-ScR guidelines to map current evidence on the interplay between sex differences, coronary artery disease, and gut microbiota. Given the limited number and heterogeneity of studies, no meta-analysis or formal assessment of certainty was performed.

### Eligibility criteria

We included observational studies with different designs (cross-sectional, case–control, prospective, and retrospective cohort studies) and intervention studies with baseline data, involving human participants. These studies focused on adults aged 18 years or older diagnosed with any form of CAD and either specifically analyzed for sex differences or included sub-analyses on sex differences from studies not originally focused on this aspect. The studies reported data on gut microbiota composition or its metabolites as an exposure. These included measures of specific bacterial taxa, microbiota diversity, ordination techniques, and the composition of gut microbiota metabolites in serum or plasma, such as TMAO, secondary bile acids, SCFAs, tryptophan, and indole derivatives. Only peer-reviewed original articles published in English were considered eligible. Two reviewers independently screened titles and abstracts, followed by a full-text review of potentially eligible studies. A single reviewer also screened the references of included articles for additional relevant material.

### Scoping review search strategies

Two reviewers collaboratively developed a comprehensive search strategy. Systematic searches were conducted in PubMed and EMBASE Library, covering all articles published during the last 10 years in these databases up to March 2025. The full search algorithms and criteria are outlined in Appendix 1 for transparency and reproducibility.

### Data extraction and evaluation of findings

Data were extracted at the study level using pre-specified forms. First, study characteristics were documented, including the year of publication, study design (observational, cohort), and demographic details for participants with CAD.

For gut microbiota data, information on composition was recorded, including diversity indices (e.g., alpha diversity, beta diversity) and taxonomic abundance at various levels (e.g., phylum, genus, species). The methods used for analysis, such as 16S rRNA sequencing or shotgun sequencing, were also noted.

Metabolite profiling, focusing on specific metabolites of interest, was documented. These metabolites included SCFA (acetate, propionate, butyrate, valerate), bile acids (primary and secondary free and conjugated bile acids, and minor bile acids reflecting microbiota isomerization activity), tryptophan metabolites (e.g., tryptophan, kynurenine, serotonin, and various indole derivatives), and choline derivatives (choline, trimethylamine, TMAO).

As this work is a scoping review, the focus was on identifying and summarizing the available and not to perform quantitative synthesis. Therefore, no formal power calculations, effect size estimations, or confidence interval analyses were conducted. Observed differences in the included studies are presented descriptively, and no inference regarding clinical significance or statistical significance across studies was attempted.

### Selected metagenomics methods

We examined the relationship between gut microbiota and CAD, with an emphasis on both the microbial composition and the functional effects arising from metabolite production. Our selection criteria favored studies that employed 16S rRNA gene sequencing and whole-genome shotgun metagenomic sequencing, as these methods offer a detailed and broad analysis of the fecal microbiota, in contrast to PCR or culture-based approaches that typically focus on specific taxa [18, 19]. Additionally, we assessed whether the studies compared bacterial diversity between individuals with CAD and healthy controls.

Alpha-diversity (α-diversity) measures the variation of gut microbiota within a single sample, considering both the richness of species and their relative abundance. Beta-diversity (β-diversity), on the other hand, evaluates the differences or similarities in microbial communities between groups, often represented using ordination plots to visualize clustering patterns. Furthermore, bacterial species were categorized according to various characteristics, such as phylogeny, metabolic functions, environmental preferences, morphology, and genetic sequence. Our synthesis of taxonomic data included both differentially abundant and discriminatory taxa.

### Selected metabolomics methods

We summarized the results from targeted and untargeted metabolomics using different mass spectrometry techniques (liquid chromatography-mass spectrometry, gas chromatography-mass spectrometry, and tandem mass spectrometry) or nuclear magnetic resonance to quantify metabolites derived from the gut microbiota resulting from our literature search [20]. We focused on metabolites directly associated with gut microbiota activity, such as SCFAs, bile acids (BAs), TMAO, and other microbial-derived metabolites.

### Definition of sex difference

In this review, we define sex as the biological attributes that distinguish individuals as male or female. These attributes typically arise from chromosomal composition, reproductive anatomy, and hormonal influences, as well as environmental or cultural factors that may affect the expression of phenotypic traits.

## Results

### Study selection

**Fig. 1:**
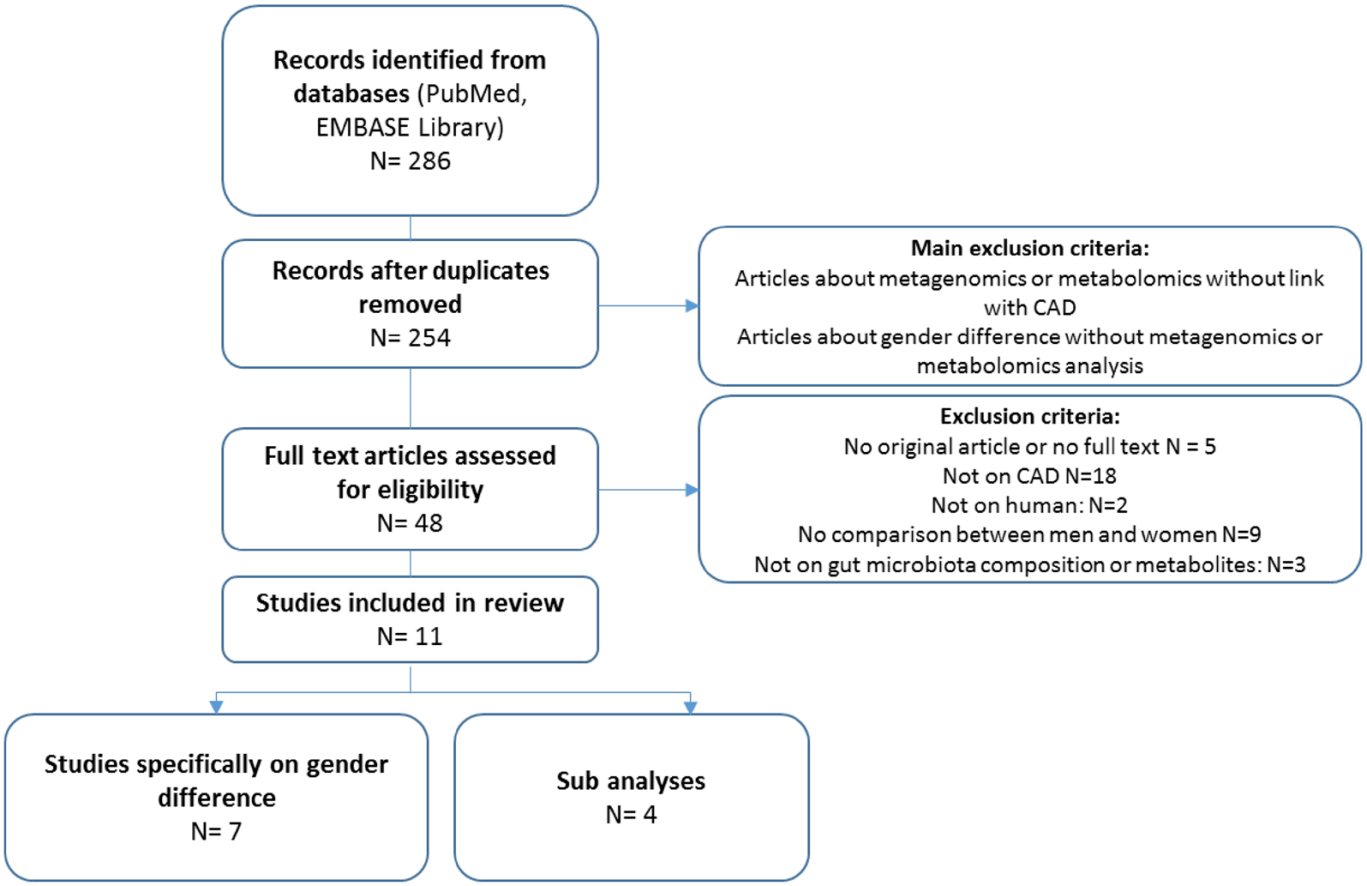
PRISMA flow chart

Our database search identified 286 potential studies, with 254 remaining after duplicate removal. Following primary screening, 48 studies were selected for full-text review (Fig. 1). Eleven studies met the inclusion criteria: 7 examined gut microbiota composition or metabolite profiles between men and women in CAD and 4 contained sub-analyses. Detailed summaries of study characteristics, results, and methodologies are presented in Table 1. The clinical characteristics are summarized in Table 2.

**Table 1:** List of selected articles

Author / Year	Participants	Type of coronary artery disease	Gut microbiota analysis	Metabolite profiling technique	Purpose of the study	Main findings	Microbiota results	Metabolomics results
Lee et al. (2015) [43]	70 (35 men, 35 women) **Subanalysis**	Acute myocardial infarction	None	Ultra-performance liquid chromatography/quadrupole time-of-flight mass spectrometry (betaine, bile acids)—untargeted	To identify sex-specific metabolic patterns in polar metabolites in serum from healthy individuals and patients with myocardial infarction	Betaine level was found higher in men with acute myocardial infarction while glycocholic acid level, a conjugated primary bile acid was found higher in women	None	Amino acids, acylcarnitines, and purines differed significantly between male and female patients with myocardial infarction
Barayani et al. (2022) [40]	56 (45 men 11 women	Acute myocardial infarction	None	High-performance liquid chromatography with tandem mass spectrometry (TMAO)—targeted	To determine whether there are sex-specific differences in TMAO concentrations before and after cardiac rehabilitation in acute myocardial infarction patients	After acute myocardial infarction, women have significantly higher levels of TMAO than men which decrease with the start of the rehabilitation	None	Female patients had significantly higher TMAO levels within the first day after hospital admission due to acute myocardial infarction. These initially high TMAO levels remained almost unchanged in the female patients until the start of cardiac rehabilitation and only reached the lower TMAO levels observed in the male patients after rehabilitation
Liu et al. (2022) [86]	219 (179 men, 40 women) **Subanalysis**	Acute myocardial infarction	None	Nuclear Magnetic Resonance spectroscopy (acetate)—untargeted	To assess the metabolic perturbations and sex and age specific metabolic characteristics associated with acute myocardial infarction	Two different gut microbiota metabolites were identified as biomarkers predicting acute myocardial infarction in men and women, respectively acetate and succinate	None	Acetate is the male-specific differential metabolites in acute myocardial infarctions with preexisting cardiovascular disease and succinate is the female specific differential metabolite in acute myocardial infarction events
Zhou et al. (2022) [46]	64 (49 men, 15 women) **Subanalysis**	Acute myocardial infarction	None	High-performance liquid chromatography with tandem mass spectrometry (TMAO)—targeted	To assess wether the occurrence of intrastent restenosis might be associated with TMAO levels	TMAO level is lower in women with intrastent restenosis and acute coronary syndrome	None	TMAO level was significantly negatively correlated with female sex (r = –0.39, P = 0.03)
Garcia-Fernandez (2024) [21]	375 (270 men, 105 women)	All types of coronary artery disease	16S RNA sequencing (Illumina MiSeq platform)	None	To evaluate the variations in the intestinal microbiota between men and women afflicted with CHD matched with control group	A sex-specific dysbiosis in the intestinal microbiota linked to CHD was observed	Men with CAD had a distinct microbial profile characterized by specific taxa like *Clostridia_UGC 14*, and *Prevotella*, while women showed an enrichment in *Actinobacteriota* and *Bifidobacteriales*	None
Bay et al. (2024) [61]	177 (89 men, 88 females)	All types of coronary artery disease	None	Mass spectrometry coupled with ultrahigh pressure liquid chromatography system (bile acids)—targeted	To compare lipidomic and bile acid profiles in the blood of patients with and without CAD stratified by sex	Altered bile acid composition was observed in men with CAD but not in women with CAD	None	Women with CAD had no differences in bile acids profiles compared to controls. Male with CAD had decreased concentrations of secondary bile acids
Couch et al. (2024) [62]	731 (346 men, 385 women)	All types of coronary artery disease	None	High‐performance liquid chromatography (tryptophan and bile acids)—targeted	To determine if sex associated metabolites are associated with incident stroke, incident coronary heart disease, hypertension and chronic kidney disease	Indole‐3‐lactic acid,and glycocholic acid were higher in male participants	None	Specific sexual dismorphism of the metabolome may contribute to sex differences in coronary artery disease
Sun et al. (2024) [45]	858 (402 men, 456 women)	Stable coronary artery disease	None	Liquid chromatography tandem mass spectrometry (TMAO)—targeted	To explore the sex and age differences in the relationship between plasma TMAO and CHD risk and severity	Plasma TMAO is significantly positively associated with the risk and severity of CHD in Chinese men	None	Plasma TMAO was higher in CAD men ≥ 65 years old and associated with a higher risk of multi-vessel disease in male patients with CHD (OR = 1.65, 95% CI: 1.18–2.32, P = 0.004), but not in females
Adhikari et al. (2024) [47]	60 (42 men, 18 women) **Subanalysis**	Stable coronary artery disease	None	Liquid chromatography tandem mass spectrometry (TMAO)—targeted	To assess biomarkers as risk factors in patients with CAD	No difference was found between men and women in terms of TMAO level < 5umol/L	None	71,4% of men and 72,2% of women have a level of TMAO < 5umol/L (p = 0,095)
Lee et al. (2024) [80]	373 (257 men, 116 women)	Stable coronary artery disease	None	Ultra Performance Liquid Chromatography System (p-cresylsulfate and indoxyl sulfate)—targeted	To examine the relationship between total p-cresylsulfate and indoxyl sulfate levels and central obesity in patients with stable	p-cresylsulfate and indoxyl sulfate levels correlate with central obesity in male CAD patients but not in females	None	Significant positive correlations were found between total p-cresylsulfate and indoxyl sulfate with obesity parameters in men with CAD but not in women with CAD
Garcia-Fernandez (2025) [44]	679 (567 men, 112 women)	All types of coronary artery disease	None	High‐performance liquid chromatography mass spectrometry (TMA and TMAO)—targeted	To evaluate differences in TMA and TMAO between men and women with CAD	CAD men have augmented TMAO levels compared with CAD women, presumably as a consequence of higher rate of TMA to TMAO oxidation. These sex differences are not observed in a non-CAD population	None	Higher TMAO levels and TMAO/TMA ratio were found in CAD men than CAD women

**Table 2:** Clinical characteristics of the selected studies

Author/Year	N		Age (years)						Male gender (%)		BMI (kg/m2)			
	Control	CAD	Control			CAD			Control	CAD	Control	CAD		
			Men	Women	p	Men	Women	p				Men	Women	p
Garcia-Fernandez et al. (2024)	329	679	59.2 ± 0.6	59.6 ± 0.9	0.711	59.1 ± 0.4	62.8 ± 0.8	** < 0.001**	242 (73,6%)	567 (83,5%)	29.3	31.1 ± 0.2	31.2 ± 0.5	0.821
Baranyi et al. (2022)	NA	56				58 ± 12,7	57,6 ± 9	0.925		45 (80,4%)		27,79 ± 3,46	27,58 ± 4,31	0.874
Sun et al. (2024)	429	429	59.9 ± 11.3	65.8 ± 8.3	0.753	60.3 ± 11.5	67.1 ± 8.3		201 (46,9%)	201 (46,9%)	26.2 ± 3.7	25.9 ± 3.3	26.0 ± 3.9	
Garcia-Fernandez et al. (2025)	375	1002	59.3 ± 0.5	59.4 ± 0.9	0.93	59.1 ± 0.3	61.8 ± 0.6	** < 0.001**	270 (72%)	827 (82,5%)	29.4 ± 0.2	31.0 ± 0.1	31.9 ± 0.4	**0.018**
Zhou et al. (2022)	NA	64								49 (76,6)				
Adhikari et al. (2024)	NA	60				50.5 (45–54)	50.0 (45–57)			42 (70)				
Bay et al. (2024)	89	88	69,2 (59,8–76,8)	67,6 (58,3–75,3)		71.9 (65.7, 77.2)	73.5 (64.1, 78.2)	0.17		44 (50)	25.45	26.3 (24.1, 28.3)	26.6 (23.5, 30.8)	0.31
Couch et al. (2024)	NA	731				66.18 ± 12.11	67.08 ± 12.57			346 (47,3)		28.51 ± 5.11	29.06 ± 6.53	
Lee et al. (2024)	NA	373				69.6 ± 12.5	73.8 ± 11.5	**0.002**				25.1 ± 3.9	24.7 ± 4.6	
Liu et al. (2024)	228	207	56.37 ± 10.45			60,6 ± 12,05			128 (56.1)	174 (84)				
Lee et al. (2015)	68	68	65.0 (58.0–70.0)	64.0 (60.0–70.5)	0.946	65.0 (56.0–71.0)	68.0 (58.0–71.0)	0.443	35 (51,5)	35 (51,5)		24.2 (22.4–25.8)	23.1 (19.7–25.3)	0.095

Author/Year	N		Hypertension (%)				Diabetes mellitus (%)				Hyperlipidemia (%)			
	Control	CAD	Control	CAD			Control	CAD			Control	CAD		
				Men	Women	p		Men	Women	p		Men	Women	p
Garcia-Fernandez et al. (2024)	329	679												
Baranyi et al. (2022)	NA	56		38 (84,4%)	8 (72,7%)	0.363						27 (60%)	7 (63,6%)	0.825
Sun et al. (2024)	429	429	280 (65.3)	142 (70.6)	178 (78.1)		139 (32.4)	85 (42.3)	137 (60.1)		317 (73.9)	165 (82.1)	187 (82.0)	
Garcia-Fernandez et al. (2025)	375	1002												
Zhou et al. (2022)	NA	64												
Adhikari et al. (2024)	NA	60		32 (76.2%)	14 (77.8%)			14 (33.3%)	8 (44.4%)			40 (95.2%)	15 (83.3%)	
Bay et al. (2024)	89	88	82 (92)	42 (95.5)	42 (95.5)	0.56	5 (5,6)	4 (9.1)	4 (9.1)	0.83				
Couch et al. (2024)	NA	731		252 (72.83)	274 (71.17)			66 (19.08)	65 (16.88)					
Lee et al. (2024)	NA	373		197(76.7)	96(82.8)	0.178		103(40.1)	49(42.2)	0.688		201(78.2)	66(56.9)	** < 0.001**
Liu et al. (2024)	228	207												
Lee et al. (2015)	68	68												

### Gut microbiota composition differences by sex in coronary artery disease

*Garcia-Fernandez *et al*.* examined gut microbiota alterations in a population of stable CAD who followed a strict diet, including 567 men and 112 women compared to a control group [21].

In analyzing gut microbiota diversity according to sex in CAD patients, no significant differences were observed in species richness, as assessed by alpha diversity indices. Pronounced sex-specific differences in beta diversity were observed among individuals with CAD, indicating distinct overall microbial community structures between male and female patients. Moreover, within each sex, comparisons between CAD and non-CAD individuals revealed significant shifts in beta diversity, suggesting that the gut microbiota responds to coronary artery disease in a sex-dependent manner.

Building on these community-level differences, taxonomic analyses reveal that sex differences in gut microbiota composition extend beyond disease states. Even in healthy individuals, men and women display distinct microbial profiles, particularly at the genus and family levels, suggesting an intrinsic influence of sex on microbial ecology [21]. In non-CAD individuals, women exhibit greater abundance of taxa such as *Bilophila, UCG_010,* and *Erysipelotrichaceae*, while men are enriched in *Alistipes, Barnesiella, Ruminococcus*, and numerous taxa from *Clostridia*. Notably, these sex-based differences become more pronounced in the context of CAD. In CAD patients, women display a microbiota enriched in beneficial or commensal genera including *Bifidobacterium, Parabacteroides*, and *Barnesiella*, whereas men show broader and deeper dysbiosis marked by increased levels of pro-inflammatory genera such as *Prevotella, Clostridia_UCG_014, Bilophila*, and *Eubacterium_siraeum_*group. Data modeling using random forest classifier further identified seven bacterial taxa that were particularly discriminant between CAD men and women: *UBA1819* (Ruminococcaceae), *Bilophila, Subdoligranulum, Phascolarctobacterium, Barnesiellaceae, Ruminococcus*, and *Ruminococcaceae incertae sedis*. Among these, *Ruminococcus* was particularly significant in distinguishing between CAD and non-CAD individuals in both sexes.

These compositional differences are relevant to CAD because many of these taxa are directly linked to inflammatory and immune pathways implicated in atherosclerosis. For example, *Prevotella* and *Bilophila* [22, 23], more abundant in men, have been associated with increased pro-inflammatory signaling and disruption of gut barrier integrity, which can promote systemic inflammation and contribute to plaque development and instability. *Prevotella* species are considered pathobionts with strong pro-inflammatory potential. They can drive mucosal and systemic inflammation by activating Th17 immune responses [24], stimulating Toll-like receptor 2, and inducing cytokines such as IL-1, IL-6, IL-8, and IL-23 from immune and epithelial cells. *Prevotella*-mediated inflammation promotes neutrophil recruitment and systemic dissemination of inflammatory mediators [25], linking increased abundance of these bacteria to chronic inflammatory conditions and potentially contributing to vascular inflammation relevant for CAD[26]. Similarly, some *Clostridia* species expanded in men can produce molecules that drive oxidative stress and inflammatory responses in the vasculature [27, 28]. In contrast, taxa enriched in women, such as *Bifidobacterium*, *Ruminococcus*, *Barnesiella*, and *Parabacteroides*, are generally considered protective: they support gut barrier function, reduce systemic inflammation, and are associated with lower oxidative stress in observational studies. *Bifidobacterium* species are beneficial commensals with anti-inflammatory and immune-modulating properties [29]. They promote immune homeostasis by upregulating regulatory T cells, maintaining intestinal barrier integrity, and modulating dendritic cell and macrophage activity, while dampening Th2 and Th17 inflammatory programs [30]. Depletion of *Bifidobacterium* is associated with impaired immune regulation and dysbiosis [31], whereas their presence supports gut and systemic homeostasis[32], which may protect against chronic inflammation relevant to CAD. Table 3 summarizes the key bacterial taxa, their sex-specific associations, and their potential mechanistic roles in CAD.

**Table 3:** Key gut bacterial taxa, sex-specific associations, and their potential mechanistic roles in coronary artery disease

Bacteria / Taxa	Sex association	Key metabolites	Potential Role in CAD / Mechanism	References
Prevotella	Men	TMA → TMAO	High TMA producer; increases intestinal permeability; promotes pro-inflammatory cytokines; may drive macrophage-rich, inflammatory plaques	[24–26]
Clostridia (general / UCG group)	Men	Indoxyl sulfate	Uremic toxin from tryptophan metabolism; impairs endothelial/smooth muscle viability; promotes apoptosis and plaque formation via miR-34a/Notch1 signaling	[27, 28]
Erysipelotrichaceae	Men	Possibly SCFA/TMA-related	Associated with pro-inflammatory responses; may exacerbate CAD progression	[97]
Eubacterium_siraeum_group	Men	SCFAs	Supports barrier integrity; modulates immune responses; reduces systemic inflammation	[158]
Bilophila	Men	LPS, secondary bile acid metabolites	Promotes gut inflammation; linked to increased intestinal permeability; may drive systemic inflammation and atherosclerosis; associated with Western diet patterns	[22, 23]
Bifidobacterium	Women	Secondary bile acids, SCFAs, reduces TMAO	Maintains gut barrier; promotes anti-inflammatory pathways; facilitates secondary bile acid production, which activates TGR5/FXR receptors; may reduce plaque inflammation	[29–32]
Barnesiella	Women	SCFAs	Butyrate/SCFA → gut barrier, anti-inflammatory, metabolic benefits	[159]

Several bacterial taxa highlighted in our sex-specific analysis of CAD patients have also been associated with cardiovascular outcomes in human studies. *Lactobacillus*, although not sex-specific in our review, has been shown to predict both disease severity and MACE in ACS patients, with higher levels associated with lower risk of all-cause death and major adverse cardiac events [33]. *Prevotella* and *Bilophila*, enriched in men with CAD in our cohort, are pro-inflammatory taxa that may contribute to systemic inflammation and plaque instability, consistent with observations linking gut dysbiosis and microbiota-derived metabolites such as deoxycholine acid (DCA) to major adverse cardiovascular events (MACE) risk in Acute ST-Segment Elevation Myocardial Infarction (STEMI) patients [34]. While other taxa in our review (e.g., *Bifidobacterium, Ruminococcus, Barnesiella, Parabacteroides*) are generally considered protective and anti-inflammatory, their presence aligns with findings from the CORDIOPREV study where specific microbiota profiles were predictive of new MACE events in CAD patients [35]. Finally, although subgingival oral microbiota biomarkers were also linked to secondary cardiovascular events, these findings highlight the broader relevance of microbial composition to cardiovascular prognosis, supporting the mechanistic plausibility of sex-specific gut microbial contributions to CAD outcomes [36].

Thus, sex-specific gut microbial composition provides a plausible mechanistic link between biological sex and CAD phenotypes. Men’s microbiota appears skewed toward taxa that promote inflammation and oxidative stress, potentially exacerbating atherogenesis, whereas women’s microbiota is enriched in taxa that may mitigate these processes, representing a protective ecological pattern. These microbiota-driven differences may act alongside hormonal and immune factors to shape the sex-specific trajectory of CAD, highlighting the microbiome as a potential mediator of cardiovascular risk differences between men and women.

### Sex-specific differences in gut microbiota-derived metabolites in coronary artery disease

- Trimethylamine-N-oxide levels are elevated in men with CAD

TMAO is a bioactive metabolite derived from dietary sources such as phosphatidylcholine, carnitine, and betaine through gut microbial metabolism. Intestinal microbiota convert these precursors into trimethylamine (TMA), which is subsequently oxidized in the liver by flavin monooxygenases (FMO1 and FMO3) to form TMAO [37]. Elevated plasma levels of TMAO have been strongly associated with an increased risk of cardiovascular diseases (CVD), including atherosclerosis [38], myocardial infarction, and stroke. Notably, studies have demonstrated a prognostic link between TMAO and CAD, showing that higher TMAO concentrations predict major adverse cardiovascular events, independent of traditional risk factors [39].

Six studies have examined sex-specific differences in TMAO levels and their potential association with CAD. Baranyi et al. [40] investigated patients with AMI and found that women had significantly higher TMAO levels than men at hospital admission. These levels remained stable in women until the start of cardiac rehabilitation, while men had lower and relatively stable TMAO concentrations throughout. Sex-specific differences in hepatic enzyme expression, particularly higher flavin-containing monooxygenase 3 (FMO3) activity in females, may explain the increased TMAO production observed in women compared to men, as supported by animal studies showing persistent FMO3 expression in females but post-pubertal downregulation in males [41, 42]. In these patients, by the end of cardiac rehabilitation, TMAO levels in women declined to levels comparable to those observed in men, indicating that lifestyle interventions may attenuate early cardiovascular risk in women through modulation of microbial metabolite production. In addition, Lee et al. [43] reported that betaine levels, a TMAO precursor, were significantly higher in men with AMI, suggesting that substrate availability may also influence TMAO metabolism in a sex-specific manner.

In contrast to the acute setting, two studies of stable CAD consistently report higher circulating TMAO levels in men, reinforcing a sex-specific pattern that may be shaped by chronic microbial and metabolic differences. Garcia-Fernandez et al. [44] showed that men with CAD had significantly higher plasma TMAO levels and a greater TMAO/TMA conversion rate compared to women with CAD. Elevated TMAO levels in men were associated with increased carotid atherosclerosis and an altered gut microbiota composition favoring TMA-producing bacteria. TMA levels were similar between men and women with CAD. Non-CVD individuals showed no sex-related differences in TMA, TMAO, or the TMAO/TMA ratio. Sun et al. [45] found that high TMAO levels were significantly associated with CAD risk in older men (≥ 65 years) but not in younger men or in women of any age compared to the control group. No difference in TMAO level is noted between healthy men and CAD men or healthy women and CAD women. Additionally, in male CAD patients, elevated TMAO levels correlated with more severe CAD, suggesting that men, especially older men, are more vulnerable to its adverse cardiovascular effects.

Further nuance is provided by two smaller sub-analyses, which offer additional insights into the sex-specific dynamics of TMAO levels in CAD. Zhou et al. [46] reported a significant negative correlation between TMAO levels and female with CAD (r = –0.39, P = 0.03), reinforcing the observation that women generally have lower TMAO concentrations. However, Adhikari et al. [47] found no significant sex differences in TMAO levels among younger CAD patients regardless of the sex, suggesting that sex-related differences in TMAO may become more pronounced with age and disease progression.

Microbial determinants of TMA production differ by sex [48]. TMA formation depends on bacterial taxa expressing TMA-generating enzymes (cutC/D, cntA/B, yeaW/X), predominantly found in *Enterobacteriaceae, Proteobacteria, Clostridia*, and related genera [49]. Men with stable CAD consistently exhibit higher abundance of these TMA-producing taxa, whereas women tend to have higher levels of anti-inflammatory, barrier-supporting commensals such as *Bifidobacterium* [50]. These compositional differences may underlie the higher chronic TMAO concentrations observed in men, given the greater microbial capacity for TMA generation.

Sex hormones influence hepatic TMAO conversion [51, 52]. FMO3 expression displays marked sexual dimorphism, with higher hepatic FMO3 activity in females [53] and post-pubertal downregulation in males. In acute settings such as AMI, this higher FMO3 activity may contribute to higher TMAO levels observed in women. In contrast, during stable disease, microbial TMA production becomes the dominant driver, resulting in higher chronic TMAO levels in men despite lower hepatic FMO3 activity.

TMAO enhances inflammatory, oxidative, and thrombotic pathways relevant to CAD. TMAO promotes activation of the NLRP3 inflammasome, increases pro-inflammatory cytokine release, augments oxidative stress, impairs endothelial nitric oxide signaling, and enhances foam cell formation by upregulating lipid scavenger receptors and disrupting cholesterol homeostasis [54–56]. It also increases platelet reactivity and thrombogenic signaling [57, 58]. These pathways may be more strongly expressed in men, who typically exhibit higher baseline inflammation, greater visceral adiposity, reduced antioxidant capacity, and higher platelet activation, potentially amplifying the pathogenic effects of TMAO. Clinical evidence indicates that higher plasma TMAO levels are associated with adverse cardiovascular outcomes. In community cohorts, such as the Multi-Ethnic Study of Atherosclerosis (MESA), elevated TMAO predicted incident atherosclerotic cardiovascular disease events over long-term follow-up, independent of traditional risk factors [59]. In patients with established CAD, TMAO levels correlate with plaque vulnerability and rupture [8], and higher concentrations are linked to increased risk of MACE during prospective follow-up [38].

Protective commensals more prevalent in women may modulate TMAO-related risk. Higher abundance of Bifidobacterium and other barrier-stabilizing taxa in women may support immune regulation, maintain epithelial integrity, and limit systemic inflammation, thereby attenuating the downstream impact of TMAO even when circulating levels are similar. Such compositional patterns may contribute to sex-specific modulation of TMAO-associated vascular risk.

- Secondary bile acids and glycocholic acid levels, a conjugated primary bile acid, are decreased in men with CAD

Bile acids, synthesized in the liver from cholesterol and further metabolized by gut microbiota, play a crucial role in lipid metabolism, inflammation, and cardiometabolic health. Alterations in BA metabolism have been linked to CAD, with some BAs exerting anti-atherosclerotic effects [60].

Bay et al. [61] investigated sex differences in lipidomic and bile acid profiles in CAD patients and found that while women with CAD showed no significant differences in bile acid concentrations compared to healthy women, men with CAD had decreased levels of secondary bile acids, such as glycolithocholic and lithocholic acids compared to healthy men. Similarly, Couch et al. [62] reported lower levels of glycocholic acid, a conjugated primary bile acid, in men with CAD compared to women with CAD. No control group was available for comparison. In the same direction, Lee et al. [43] found that glycocholic acid was elevated in women with AMI.

Secondary BAs such as lithocholic acid (LCA) and DCA, reduced in men with CAD, are primarily produced by gut microbial transformation of primary bile acids. Unconjugated bile acids (like cholic acid (CA), chenodeoxycholic acid (CDCA), DCA, hydrodeoyxcholic acid (HDCA)) are more potent ligands for Farnesoid X Receptor (FXR) and Takeda G protein-coupled receptor 5 (TGR5) and can directly modulate cholesterol metabolism, inflammation, and vascular signaling. Conjugated bile acids (glyco- or tauro-conjugates) are less potent at FXR/TGR5 but can facilitate bile acid solubility, enterohepatic circulation, and lipid absorption. Lower secondary bile acid levels may therefore reflect reduced microbial conversion capacity in men, consistent with known depletion of microbial diversity and metabolic activity in cardiometabolic disease [63]. Women with CAD do not show comparable reductions; this may reflect a more preserved microbial environment or differences in bile acid flux through classical versus alternative hepatic synthetic pathways.

FXR governs bile acid synthesis via feedback inhibition of CYP7A1 (Cytochrome P450 Family 7 Subfamily A Member 1) and CYP8B1 (cytochrome P450, family 8, subfamily B, polypeptide 1) and modulates cholesterol metabolism, inflammatory signaling, and enterohepatic circulation [64]. Given that CDCA is the most potent endogenous FXR agonist, relative increases in CDCA-rich bile acid pools reinforce FXR activity [65], whereas reductions in secondary BAs diminish TGR5 agonism [63, 66]. In men with CAD, lower secondary BAs may shift the pool toward stronger FXR dominance and reduced TGR5 stimulation, creating an imbalance that favors impaired gut–liver signaling and heightened inflammatory susceptibility. Women, who do not show significant depletion of secondary bile acids, may maintain a more balanced FXR–TGR5 activation profile.

TGR5 activation, primarily driven by unconjugated secondary bile acids (LCA > DCA > CDCA > CA)[67], exerts beneficial effects on vascular and metabolic pathways, including GLP-1 secretion[68], enhancement of endothelial nitric oxide production, smooth muscle relaxation, and attenuation of macrophage-mediated inflammation [69, 70]. Reduced availability of these potent TGR5 agonists in men may contribute to diminished anti-inflammatory and vasoprotective signaling. By contrast, preserved levels of secondary bile acids in women could help maintain TGR5-mediated anti-inflammatory tone, potentially mitigating the adverse impact of CAD-associated metabolic stress.

Both FXR and TGR5 exert anti-inflammatory actions in immune cells, including macrophages, dendritic cells [71], and Natural killer T (NKT) cells [72]. A bile acid pool skewed toward reduced TGR5 activation may weaken anti-inflammatory signaling networks, enhancing Interleukin 6 (IL-6), Tumor Necrosis Factor α (TNF-α), and Interferon- γ (IFN-γ) activity [73]. Given the higher baseline systemic inflammation and visceral adiposity in men, diminished TGR5 signaling may amplify inflammatory responses in CAD, while more preserved BA profiles in women may maintain partially protective immunometabolic regulation.

Collectively, the sex-specific patterns observed—men with CAD presenting lower levels of secondary bile acids, women showing relative preservation or even elevation of certain conjugated bile acids—align with a mechanistic model in which men experience a shift toward reduced TGR5 agonism and altered FXR-driven feedback, contributing to a pro-inflammatory and less metabolically adaptive state. Women appear to maintain a more balanced bile acid signaling environment that may buffer against some pathogenic consequences of CAD and acute myocardial injury. Furthermore, females tend to exhibit higher secondary and conjugated bile acids, while men may have lower secondary bile acids but higher levels of certain conjugated primary bile acids depending on age and hormonal status [74–76]. Unconjugated bile acids such as CA, CDCA, DCA, and HDCA have been inversely associated with CAD risk and cardiovascular mortality [77, 78]. These sex-specific bile acid profiles, partly shaped by gut microbiota and hormonal influences, may differentially modulate FXR/TGR5 signaling, lipid metabolism, and inflammatory pathways, suggesting a potential mechanism by which men and women could experience distinct cardiovascular diseases states.

- Indoxyl sulfate, a tryptophan derivative, has positive correlation in men with CAD

Indole, a degradation product of the amino acid tryptophan, is produced by intestinal bacteria and subsequently absorbed and metabolized in the liver into IS, a key protein-bound uremic toxin. Research suggests that IS contributes to the progression of cardiovascular disease by promoting cardiac fibrosis, inducing endothelial senescence, stimulating vascular smooth muscle cell proliferation, and activating monocytes and macrophages through an oxidative stress-dependent pathway [79].

Couch et al. [62] also found that men with CAD had lower levels of indole-3-lactic acid, a microbial metabolite derived from tryptophan metabolism than women with CAD. Lee et al. [80] explored the relationship between gut-derived uremic toxins and central obesity in CAD patients. They found significant positive correlations between IS, a tryptophan derivative, with measures of central obesity (e.g., waist-to-hip ratio, conicity index, adiposity body shape index) in men with CAD but not in women with CAD. Another study [43] also found that IS levels were lower in CAD patients of both sexes, with a more pronounced reduction in men; however, this sex difference did not reach statistical significance. Since tryptophan derivatives have been implicated in inflammation and cardiovascular health, these differences may contribute to sex-specific disease mechanisms.

IS promotes oxidative stress (via NADPH Oxidase 4 (NOX4) and mitochondrial reactive oxygen species (ROS)), impairs endothelial repair and endothelial nitric oxide synthase activity, activates aryl hydrocarbon receptor (AHR)/NF-κB signaling in monocytes/macrophages, induces pro-inflammatory cytokine release, enhances macrophage foam-cell formation, stimulates vascular smooth muscle cells osteogenic transformation, and increases thrombogenic signaling (tissue factor expression, platelet-leukocyte interactions) [81]. In men—who characteristically display greater visceral adiposity, higher baseline inflammatory tone, and a gut microbiota profile enriched in pro-inflammatory taxa—the association between IS and central obesity may amplify local and systemic oxidative/inflammatory cascades that accelerate atherogenesis, plaque vulnerability, calcification, and thrombosis [81, 82]. By contrast, women in the previously described cohorts tended to retain higher levels of certain indole derivatives and/or have more barrier-supporting commensals (e.g., *Bifidobacterium*), which could limit intestinal translocation of indole precursors or modulate host responses to IS, thereby attenuating IS-driven vascular injury. Importantly, renal function, disease stage and age modulate circulating IS and may account for some inconsistencies across studies [82].

Clinical evidences show that in patients with stable angina, higher serum IS levels were independently associated with more severe coronary stenosis, higher Agatston calcium scores, modified Gensini scores, and greater numbers of diseased vessels, suggesting a correlation with CAD burden [83]. In acute coronary syndrome (ACS) patients undergoing primary percutaneous coronary intervention, elevated IS levels predicted six-month composite events—including death, myocardial infarction, heart failure hospitalization, and bleeding events—with a hazard ratio of 10.6 (95% CI: 1.63–69.3), independent of conventional risk factors [84]. IS has significant prognostic utility in CAD and ACS, reinforcing its potential role as a clinically relevant biomarker of adverse cardiovascular outcomes.

Overall, the scoping data support a hypothesis in which sex-specific differences in microbial tryptophan metabolism—interacting with adipose distribution and baseline immune state—render men more vulnerable to the pro-oxidative, pro-inflammatory and pro-thrombotic effects of IS, thereby contributing to greater CAD burden and severity in men versus women.

- Acetate, a SCFA, and histidine are enriched in men with AMI and preexisting cardiovascular disease

SCFA are microbial-derived metabolites produced through the fermentation of dietary fibers by gut bacteria. The most abundant SCFAs include acetate, propionate, and butyrate, which play essential roles in host metabolism, immune regulation, and cardiovascular health [85].

Liu et al. [86] identified sex-specific differences in gut microbiota-related metabolites in AMI patients. Succinate, a fermentation byproduct of dietary fibers, was a female-specific metabolite in AMI, whereas acetate, a SCFA, was male-specific in AMI patients with pre-existing cardiovascular disease.

Our scoping analysis identified acetate as a male-enriched SCFA in AMI patients with pre-existing cardiovascular disease. This observation may have bidirectional mechanistic implications for sex differences in CAD. On one hand, acetate signaling via the G-protein–coupled receptor GPR43 activates AMPK in plaque macrophages, suppresses macrophage proliferation, reduces ROS production, and limits pro-inflammatory cytokine expression—actions that reduce plaque formation and progression in experimental atherosclerosis models [87]. On the other hand, acetate can be metabolically converted to acetyl–CoA within endothelial cells, enhancing protein acetylation of TGF-β pathway components (Activin receptor-like kinase 5, intracellular mediator of TGF-β/ALK5 signaling (SMADs)) via Acyl-CoA synthetase short-chain family member 2 (ACSS2)-dependent pathways and thereby stabilizing TGF-β signaling and promoting endothelial-to-mesenchymal transition (EndMT), a driver of vascular fibrosis and chronic vascular remodeling [88]. Additionally, acetate-related signaling has been linked to modulation of endothelial pyroptosis pathways through histone deacetylase–related mechanisms in preclinical studies [89]. Taken together, the enrichment of acetate in men with AMI and established CVD may reflect a context-dependent balance: in some vascular cell compartments (macrophages) acetate may exert anti-atherogenic, anti-inflammatory effects, whereas in endothelial metabolic states favoring ACSS2 activity it may promote profibrotic EndMT and maladaptive remodeling. Sex-specific factors—differences in gut microbial composition, substrate availability, endothelial metabolic programming, and hormonal regulation—could shift this balance toward net benefit or harm. Thus, higher acetate in men could contribute both protective and deleterious pathways, potentially explaining part of the sex-specific phenotype in CAD depending on disease stage and cellular context.

While our current data do not specifically report sex-stratified levels of these metabolites in CAD or AMI, emerging literature suggests that sex differences in butyrate-producing bacteria exist and may have cardiometabolic implications. For instance, women have been shown to harbor a higher relative abundance of butyrate-producing genera such as *Oscillospiraceae* and *Lachnospiraceae*, along with enriched genes for butyrate synthesis across multiple pathways, compared with men [90]. In animal and human in vitro studies, male microbiotas exhibited lower butyrate production and associated metabolic dysfunction, including increased triglycerides, leptin, and oxidative stress, suggesting sex-specific effects on lipid metabolism [91, 92].

However, these metabolites were not found to be biomarkers to classify between controls and AMI patients. Histidine, a semiessential amino acid with antioxidant and anti-inflammatory properties, also emerged as male-specific in AMI events, and alterations in histidine metabolism were more pronounced in patients with concomitant CVD. Notably, histidine metabolism may be linked to the generation of downstream metabolites such as histamine, glutamate, and glutamine, which have been implicated in AMI pathophysiology [93, 94]. In addition, the microbial metabolite imidazole propionate—derived from histidine rather than fiber fermentation—has been identified as a potential independent risk factor for atherosclerosis, highlighting the need for further studies evaluating its sex-specific effects on CAD [95].

**Fig. 2:**
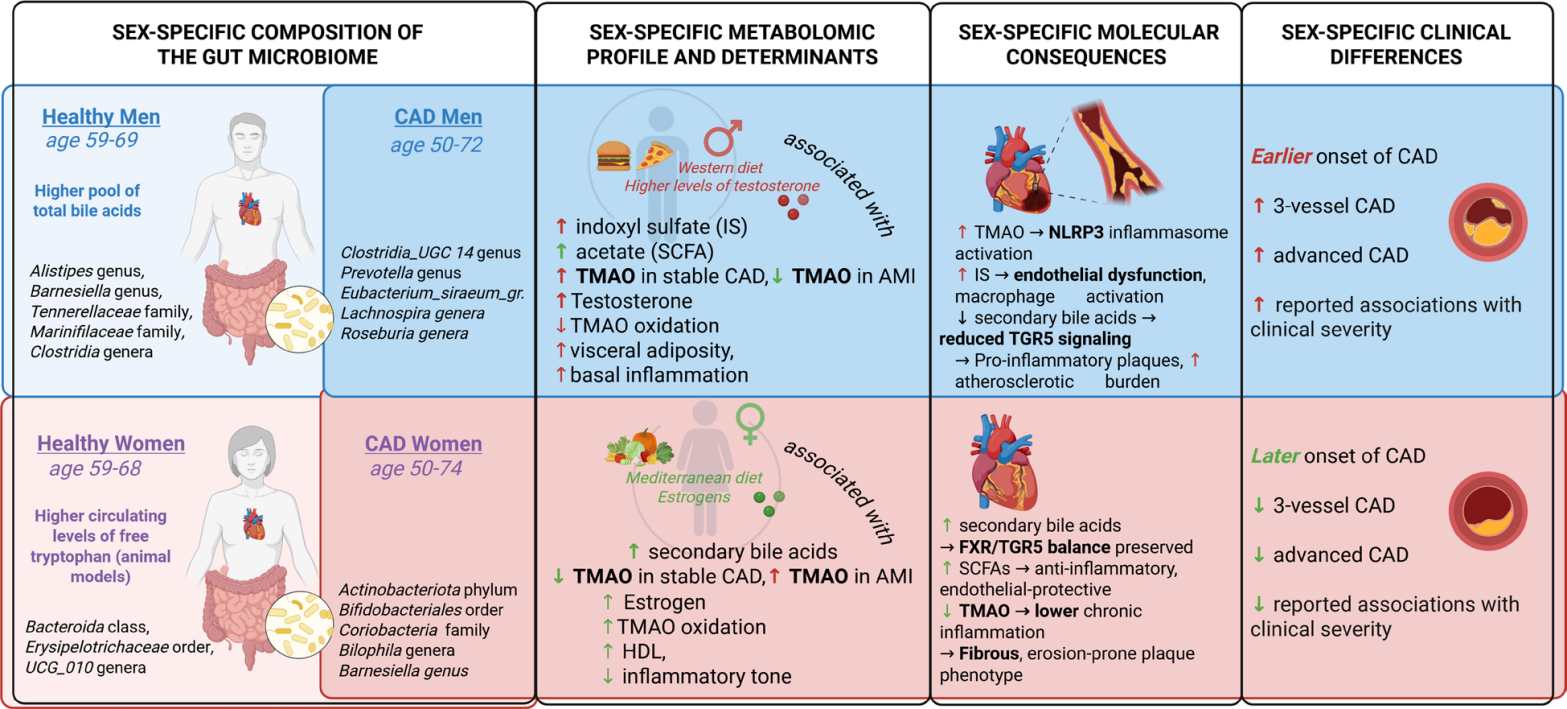
Sex-specific composition of the gut microbiome, metabolomic profiles, potential molecular consequences, and clinical phenotype in coronary artery disease (CAD). This figure summarizes currently reported sex differences across four domains: (1) microbiome composition in healthy individuals and patients with CAD, (2) metabolomic patterns and upstream determinants (dietary patterns, sex hormones, visceral adiposity, inflammation), (3) molecular and vascular consequences of microbial metabolites, and (4) characteristic clinical features. Men with CAD tend to exhibit higher TMAO and indoxyl sulfate, lower secondary bile acids, increased NLRP3-driven inflammation, and more extensive atherosclerosis. Women show higher secondary bile acids, lower TMAO in stable CAD (but not AMI), preserved FXR/TGR5 signaling, and lower chronic inflammatory tone, together with later and less severe CAD presentation. These patterns are derived from observational studies and should be interpreted as associative rather than causal. Sex differences illustrated in this figure likely reflect the combined influence of hormonal, genetic, and immune factors on gut microbiota composition and microbial metabolism, which are discussed in detail in the text. This figure was made with Biorender.com

The Fig. 2 is a comprehensive summary of the results and illustrates the interconnected sex-specific differences observed across the gut microbiome, metabolomic signatures, molecular pathways, and clinical expression of coronary artery disease (CAD). In men, both healthy individuals and those with CAD display a gut microbial pattern enriched in bile-acid–associated taxa (such as *Alistipes, Barnesiella*, and *Clostridia*), accompanied by higher circulating bile acids, elevated indoxyl sulfate, acetate, visceral adiposity, and a pro-inflammatory metabolic profile driven by Western-diet patterns and higher testosterone levels. These features converge mechanistically through enhanced TMAO production, NLRP3 inflammasome activation, impaired TGR5 signaling, and endothelial dysfunction—translating clinically into an earlier onset of CAD, more frequent multivessel disease, and higher in-hospital mortality during acute myocardial infarction. In contrast, women, both healthy and with CAD, show enrichment in microbial taxa linked to tryptophan metabolism and secondary bile-acid production, alongside higher estrogen levels, preserved TMAO oxidation, lower inflammatory tone, and cardioprotective SCFA-related pathways. These biological differences align with a later onset of CAD in women, a lower prevalence of advanced or three-vessel disease, and reduced in-hospital mortality in acute events. Together, the figure summarizes how microbiome composition, hormonal environments, and metabolite profiles interact to shape distinct sex-specific trajectories of CAD development and severity.

## Discussion

This review highlights possibility of biologically sex-specific differences in gut microbiota composition and microbial metabolite profiles among individuals with CAD. Although current evidence remains limited, emerging data suggest that men and women with CAD may harbor distinct gut microbial profiles and exhibit sex-specific patterns in microbiota-derived metabolites such as TMAO, IS, secondary BAs, and SCFAs. These observations point toward potential sex-dependent interactions between host physiology, microbial metabolism, and immune responses along the gut–heart axis. While causality has yet to be firmly established, these findings raise important questions about the role of gut microbiota in shaping sex differences in CAD risk and progression. The very limited number of sex-stratified microbiome studies in CAD represents a major knowledge gap in the field. The scarcity of evidence is not only a limitation of current literature but also a key finding of this review, underscoring the need for dedicated, adequately powered investigations.

The results of the reviewed studies highlight sex-based differences in gut microbiota composition and metabolites in CAD with (1) in terms of microbiota composition, men with CAD had a distinct microbial profile characterized by specific taxa like *Clostridia_UGC*, *Erysipelotrichaceae* and *Prevotella*, while women showed an enrichment in *Actinobacteriota* and *Bifidobacterium*, (2) in terms of gut metabolites, men exhibit higher levels of TMAO, IS and acetate, while women had higher levels of secondary bile acids and lower levels of TMAO. Because direct microbiota data remain extremely scarce, integrating complementary evidence from microbial metabolites, hormonal regulation, host genetics, and plaque biology was essential to construct a biologically plausible framework for sex-specific interactions along the gut–heart axis.

Among the taxa enriched in the gut microbiota of men with CAD, *Clostridia*_UCG_014, *Prevotella*, and members of the Erysipelotrichaceae family have been implicated in pro-inflammatory activity and compromised gut barrier integrity. *Prevotella*, in particular, has been associated with increased intestinal permeability and heightened production of pro-inflammatory cytokines, thereby potentially contributing to the initiation and progression of atherosclerosis [26, 96]. The *Erysipelotrichaceae* family has also been associated with pro-inflammatory responses [97], which could exacerbate CAD progression. *Lachnospira, Roseburia*, and *Eubacterium_siraeum_group* are known to produce SCFA, which are more prone to support gut barrier integrity, reduce inflammation, and may protect against atherosclerosis by modulating immune responses and lipid metabolism. In the gut microbiota of CAD women, *Bifidobacterium* species are considered protective due to their role in maintaining gut barrier function and modulating anti-inflammatory pathways [98]. However, this discussion relies on the single study that specifically examined gut microbiota in male and female patients with CAD. Given the high variability of the gut microbiota over time and across disease states and contexts, additional studies with larger, well-characterized cohorts are needed to corroborate these findings and to explore potential links between specific microbial taxa and sex-related differences in CAD.

The gut microbiota plays a crucial role in transforming dietary nutrients into bioactive molecules, among which TMAO has garnered significant attention for its potential role in promoting atherosclerosis [99]. TMAO is generated from TMA, a metabolite produced by the gut microbiota through the breakdown of choline and L-carnitine. Once formed, TMA is transported to the liver, where it undergoes oxidation by FMO3 [100]. The conversion of choline and carnitine to TMAO is highly dependent on the composition and balance of the gut microbiota. TMAO levels have been found to be higher in men than in women with CAD and are notably associated with the severity of atherosclerotic lesions in men. This observation raises important questions about the underlying mechanisms driving sex-specific differences in TMAO metabolism. Interestingly, the gut microbiota composition differs between men and women with CAD. Women exhibit an enrichment of *Actinobacteria* and *Bifidobacterium*, whereas men with CAD have a higher abundance of *Prevotella*. We can first hypothesize that elevated TMAO levels in men is that *Prevotella* is known to be a high potential producer of TMA [101] while *Bifidobacterium* is reported to be negatively correlated to TMAO [102]. A second explanation might rely on sex hormones. Since FMO3 is responsible for converting TMA to TMAO, higher estrogen levels in women are described to reduce FMO3 expression by suppressing FMO3 transcription via endoplasmic reticulum binding, thereby lowering TMAO production, with low concentration of estrogen already sufficient to inhibit FMO3 [103]. In the same direction, testosterone directly inhibits FMO3 activity in the liver in mice. However, the observation that hormone effects alone do not fully explain plasma TMAO differences is supported in the same study [51]. As a third explanation, we suggest that dietary factors play a significant role in the synthesis of TMAO, especially that men and women are known to have different diet tendencies: more Mediterranean diet for women and more Western diet for men [45–47]. In experiments involving both mice and human cohorts, sex differences in TMAO levels were not consistently observed. For example, plasma TMAO levels in random samples from the Genebank cohort did not show significant differences between sexes. These findings suggest that although FMO3 is essential for converting TMA to TMAO, its activity alone does not fully account for interindividual or sex-related variation. Dietary factors, particularly the intake of choline-rich foods, emerge as major modulators of TMAO synthesis, acting independently or in concert with hormonal regulation [104].

In AMI, the stress response and systemic inflammation might trigger a rapid upregulation of TMAO production in women—who, due to higher FMO3 expression, already have a more efficient conversion of TMA to TMAO. This could reflect a transient increase in TMAO as part of an acute-phase reaction or a marker of heightened platelet activation and thrombotic risk in women [41, 42]. In contrast, in stable CAD, where the disease is more chronic and potentially more influenced by diet and microbiota composition, men tend to have higher TMAO levels, possibly due to greater abundance of TMA-producing bacteria or less Mediterranean diet adherence. In this context, the chronic exposure to pro-atherogenic microbial profiles in men may be a more dominant driver of TMAO elevation than the enzymatic conversion capacity alone. So, the difference between AMI and stable CAD might reflect how acute inflammatory states and chronic microbiota-derived risk factors interact differently in men and women, depending on the disease phase.

Acetate is the most abundant SCFA in the gut, followed by propionate and butyrate [105], and has been found to be elevated in men with CAD. *Lachnospira* and *Roseburia*, both acetate-producing genera, were reported to be more prevalent in the gut microbiota of male CAD patients [106]. While acetate has been shown to exert both beneficial and detrimental effects on host metabolism [107, 108], its impact appears highly context-dependent [109]. At physiologic concentrations, acetate supports cardiovascular health by activating AMP-activated protein kinase (AMPK) in macrophages, thereby dampening oxidative stress and systemic inflammation [87, 110]. In contrast, under conditions such as a high-fat diet, excessive acetate production may drive maladaptive metabolic responses—including parasympathetic overstimulation, elevated ghrelin secretion, hyperphagia, insulin resistance, and lipid accumulation [111]. Thus, while acetate may be atheroprotective under balanced conditions, chronically elevated levels could promote metabolic dysfunction, highlighting the importance of context and host–microbiota interactions.

An increased abundance of *Clostridia* species in men with CAD may underlie some of the observed sex differences in cardiovascular disease, particularly in the development of atherosclerosis. *Clostridia* are known producers of IS, a uremic toxin derived from microbial tryptophan metabolism, which plays a critical role in vascular pathology [112]. IS has been shown to impair endothelial and smooth muscle cell viability, inhibit proliferation and migration, and promote apoptosis and plaque formation. Mechanistically, IS upregulates miR-34a, leading to downregulation of the Notch1 signaling pathway and contributing to vascular dysfunction. Elevated IS in men, potentially driven by Clostridia enrichment, may help explain their heightened susceptibility to atherosclerosis [113].

In contrast, *Bifidobacterium*, more commonly enriched in the gut microbiota of women with CAD, may confer a protective metabolic influence. While Bifidobacterium does not produce secondary bile acids directly, it facilitates their generation by deconjugating primary bile salts, which are then further metabolized by taxa such as *Eubacterium* and *Clostridium* [29]. Secondary bile acids modulate host immunity by promoting regulatory T cell (Treg) differentiation and dampening inflammatory responses [114], and may protect against atherosclerosis through activation of bile acid receptors such as TGR5 and FXR [115]. Interestingly, serum bile acid levels are typically higher in healthy men than women [116], suggesting that men with CAD may experience bile acid dysregulation that disrupts these immunomodulatory pathways.

The sex differences now well-described in CAD [117] could be partially explained by a combination of hormonal, microbiome, and metabolic factors. Significant sex differences in gut microbiota were observed across various species, particularly in *Actinobacteria, Bacteroidota,* and *Bacillota*, with men and women hosting distinct microbial populations [118]. While sex-specific differences were noted in microbial composition, such as in the abundance of *Bacteroidota* and *Bacillota*, there were no clear sex differences in overall microbiota heterogeneity or diversity. However, certain species interactions and microbial consortia (triads) showed sex-dependent patterns, highlighting the nuanced influence of sex on microbial dynamics. Sex-dependent species interactions and core microbial species suggest that men and women may have unique microbiota compositions with potential implications for health outcomes. These differences could influence cardiovascular disease risk, as the microbiota plays a critical role in lipid metabolism, inflammation, and immune regulation, all of which are involved in atherosclerosis. Women with CAD tend to be older, which could be linked to the decline in estrogen levels post-menopause [119]. Estrogen has a protective effect on cardiovascular health, including its influence on lipid metabolism, inflammation, and endothelial function. Additionally, women with CAD often present with fewer multi-vessel lesions compared to men [120, 121], which could be attributed to a protective effect of secondary bile acids, which are influenced by hormonal changes and dietary factors. These bile acids, produced via the gut microbiota’s metabolism, help modulate lipid homeostasis and inflammatory pathways, potentially leading to less severe disease in women. Moreover, women with CAD tend to have lower circulating TMAO levels, a metabolite linked to increased atherosclerosis risk, due to a different gut microbiota composition (e.g., higher *Bifidobacterium* and lower *Prevotella* abundance). This lower TMAO production might contribute to less aggressive disease progression, as TMAO is associated with increased inflammation and plaque instability. In addition, women with CAD exhibit lower severity of the CAD [122], possibly due to less pronounced inflammatory responses, which could also be influenced by estrogen’s anti-inflammatory effects. While men with CAD generally display a more pro-atherogenic microbiota and metabolite profile, postmenopausal women may experience shifts in gut microbial composition and metabolism that partially explain their higher cardiovascular mortality once disease is established. Evidence indicates that menopause is associated with reduced microbial diversity, altered abundances of taxa such as *Bacteroides, Prevotella*, and *Sutterella* [123], and changes in microbial metabolism including increased TMAO production [124] or decreased circulating SCFA [125]. These microbiota and metabolite changes, together with declining estrogen levels, may exacerbate inflammation, endothelial dysfunction, and plaque instability, contributing to higher post-CAD risk in women compared with men.

Additional inflammatory and metabolic pathways may influence sex-specific cardiovascular risk, although evidence linking them directly to CAD remains limited. Experimental human studies show clear sex differences in inflammatory responses to lipopolysaccharide (LPS). Men exhibit a stronger cytokine response to LPS administration than women, while estrogens attenuate LPS-induced macrophage activation and inflammatory gene expression [126, 127]. Similarly, circulating branched-chain amino acids (BCAAs)—which are partly regulated by gut microbial biosynthetic pathways—might display sex differences. Men show higher plasma BCAA concentrations and stronger associations with metabolic risk, influenced by androgen-driven BCAA metabolism [128]. Microbiome studies also demonstrate that specific bacterial taxa, such as Prevotella copri and Bacteroides vulgatus, contribute to elevated circulating BCAAs [129]. Emerging evidence suggests that circulating BCAAs are associated with the presence and severity of CAD. Mendelian randomization analyses indicate a potential causal relationship between higher BCAA levels and increased CAD risk, partly mediated by blood pressure and type 2 diabetes, and linked to plaque progression and thrombosis [130]. Observational studies and case–control analyses have further shown that elevated BCAAs correlate with angiographic CAD and subclinical atherosclerosis, such as increased carotid intima-media thickness, as well as traditional cardiovascular risk factors independently of diabetes, hypertension, or dyslipidemia [131, 132]. Despite these associations, there is currently no strong evidence linking sex-specific differences in BCAA levels to CAD outcomes, and exploratory studies on sex hormones and BCAA interactions have not demonstrated consistent or causal effects [133].

Genetic studies demonstrate a highly polygenic architecture of CAD [134], with sex-specific signals still underdetected due to under-representation of women and limited sex-stratified analyses [135–137]. This includes insufficient coverage of the X chromosome despite its enrichment in immune genes relevant for sex-differential vascular phenotypes [138, 139]. Sex-biased SNPs have been linked with distinct vascular remodeling pathways—IL6 and OLR1 predominantly in women [140] and PDE4D/CDKN2A in men [141]—supporting divergent mechanisms of endothelial dysfunction versus inflammatory/macrophage-driven responses [142, 143]. Plaque-level transcriptomic data reinforce these molecular differences, showing stronger smooth muscle cell phenotypic modulation and fibrous, EndMT-associated plaque features in women, and more inflammatory, immune-enriched plaques in men [144, 145].

Epigenetic regulation further modifies cardiovascular risk in a sex-dependent manner, with differential DNA methylation patterns linked to inflammation, lipid metabolism, and clinical outcomes—such as Pla2g7 promoter methylation in women or reduced global methylation in men [146–149]. Hormone–epigenome interactions, including estrogen-driven modulation of HDAC activity and restoration of SIRT1, add another regulatory layer shaping sex-specific vascular responses [150, 151]. Perturbations in chromatin accessibility and enhancer–promoter interactions have also been linked to sex-biased cardiac phenotypes, underscoring the role of epigenetic architecture in sex-specific gene expression [152–154].

**Fig. 3:**
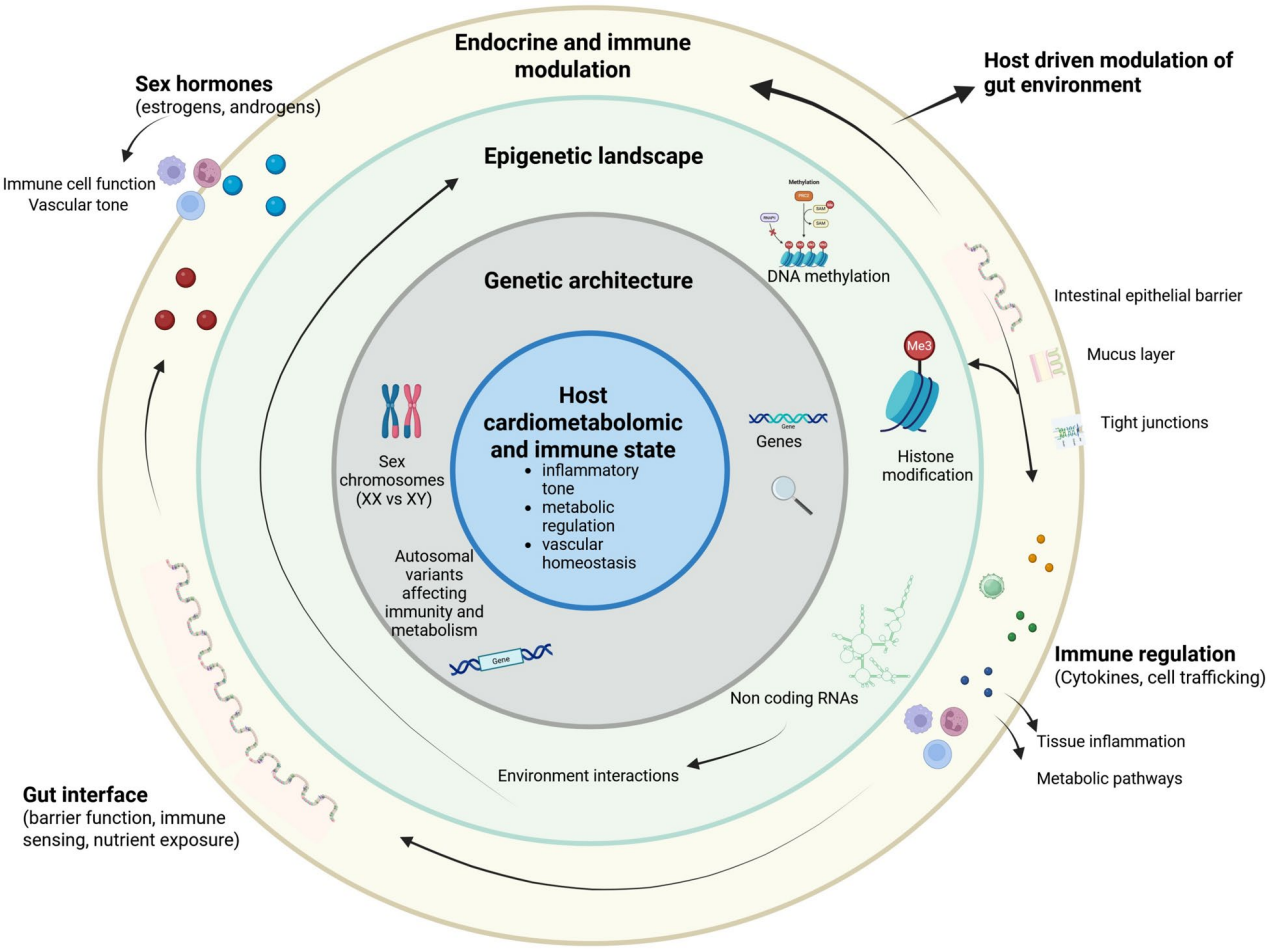
Host-level biological factors shaping sex-specific responses along the gut–heart axis. This conceptual, hypothesis-generating figure illustrates host-level biological layers that may contribute to sex-specific responses along the gut–heart axis in coronary artery disease. At the core, host cardiometabolic and immune state—encompassing inflammatory tone, metabolic regulation, and vascular homeostasis—is shaped by genetic architecture, including sex chromosomes (XX vs. XY) and autosomal variants influencing immune and metabolic pathways. These genetic effects are further modulated by the epigenetic landscape, including DNA methylation, histone modifications, and non-coding RNAs, which regulate gene expression in a sex- and context-dependent manner. Endocrine and immune modulation, driven by sex hormones (estrogens and androgens) and sex-biased immune responsiveness, further influences immune cell function, cytokine signaling, and vascular tone. The outer layer represents the gut interface as a site of host–environment interaction, including epithelial barrier integrity, mucus layer organization, tight junctions, and pattern-recognition receptor–mediated immune sensing. Bidirectional arrows indicate potential interactions between host biology and the gut environment, reflecting host-driven modulation of gut conditions and immune sensing of luminal signals. This figure does not depict specific gut microbial taxa or microbial metabolites, which are summarized separately, and is intended to highlight upstream host mechanisms that may bias sex-specific responses along the gut–heart axis rather than to imply direct causality. This figure was made with Biorender.com

These genetic and epigenetic differences converge with gut microbiota–host interactions (Fig. 3). Sex-biased immune loci and epigenetic regulation of intestinal epithelial genes influence mucosal immunity, barrier function, and antimicrobial peptide expression, thereby shaping microbial communities in a sex-dependent manner [134, 155]. Women tend to harbor higher SCFA-producing bacteria, supporting barrier integrity and lower systemic inflammation, consistent with their higher prevalence of fibrous, erosion-prone plaques [145, 156]. Men, conversely, show enrichment in pro-inflammatory and TMA/TMAO-producing taxa linked to metabolic dysfunction, oxidative stress, and macrophage-rich plaque phenotypes. Hormone–microbiota interactions—such as β-glucuronidase-mediated modulation of estrogen metabolism—provide an additional mechanistic pathway explaining sex-specific CAD patterns, especially the shift toward dysbiosis, inflammation, and increased risk after menopause [156]. While genotype exerts a strong influence on microbial structure, sex-specific inflammatory and hormonal pathways likely act as key modulators of the microbiota, contributing to the observed divergence in plaque biology and disease progression [156, 157].

### Limits

The evidence included in this review presents several limitations. First, the number of available studies is limited (n = 11), and only one study directly examined the gut microbiota in the context of CAD with consideration of sex. Most studies were descriptive in nature and did not specifically explore the mechanistic links between gut microbiota composition and sex differences in CAD pathophysiology or outcomes. The limited number of studies available makes meaningful comparisons between them challenging. The discussion on the gut microbiota composition is only based on one study. Therefore, while our findings suggest potential sex-specific microbial patterns, it remains unclear whether these differences contribute directly to CAD pathophysiology or reflect secondary effects of other sex-related factors. Future mechanistic studies are required to validate causal relationships and clarify underlying biological pathways.

Furthermore, there is considerable heterogeneity across studies in terms of population characteristics, sample size, inclusion criteria, microbiome sampling and sequencing methodologies, metabolomic platforms, and clinical endpoints. These differences limit the direct comparability of findings between studies and currently preclude meaningful meta-analyses. This heterogeneity also complicates the identification of consistent sex-specific microbial or metabolic signatures in CAD, highlighting the need for standardized protocols and larger, well-characterized cohorts in future research. A key limitation of this study is the potential influence of well-established confounders on gut microbiome composition. Factors such as diet, medications, geography, socioeconomic status, and lifestyle can substantially impact microbial profiles. Without comprehensive control for these variables, observed sex-specific differences may partly reflect underlying systematic differences between male and female populations rather than true biological variation. This limitation should be considered when interpreting the findings and highlights the need for carefully controlled studies in future research. Although direct evidence linking gut microbiota to sex differences in CAD remains limited, we have integrated mechanistic insights from genetics, epigenetics, hormone signaling, and microbial metabolism to provide a comprehensive framework supporting this hypothesis.

An additional limitation is the near-complete absence of studies evaluating whether sex-specific microbial or metabolic profiles influence CAD progression, prognosis, or clinical outcomes. None of the included studies were designed to assess longitudinal changes, event rates, or treatment response in relation to gut microbiota differences between men and women. As a result, the current evidence does not allow conclusions about whether these microbial or metabolite variations translate into measurable differences in disease trajectory or outcomes.

Accordingly, the interpretations presented here should be regarded as hypothesis-generating rather than definitive, reflecting the early stage of sex-specific microbiome research in cardiovascular disease.

## Conclusion

This review highlights the emerging, yet underexplored, role of sex-specific interactions between gut microbiota, microbial metabolites, and host biology in CAD. Preliminary evidence suggests that men and women exhibit distinct microbial compositions and metabolite profiles, with men showing enrichment in pro-inflammatory, TMA/TMAO-producing taxa such as *Prevotella* and *Clostridia*, and women harboring SCFA-producing, barrier-supporting microbiota like Bifidobacterium. These microbial patterns align with sex-specific plaque phenotypes—macrophage-rich, inflammatory lesions in men versus fibrous, EndMT-rich plaques in women. By integrating mechanistic links from host genetics, epigenetics, hormone signaling, and microbial metabolism, this review supports the hypothesis that gut microbiota may contribute to sex differences in CAD, However, while this review integrates emerging mechanistic hypotheses linking gut microbial composition, metabolites, and sex-specific coronary artery disease phenotypes, most evidence remains correlative. Current data are largely derived from cross-sectional studies, limiting causal inference. Sex-related differences in the gut microbiota may reflect not only biological determinants—such as hormonal and genetic factors—but also lifestyle, dietary, and pharmacological influences. Future longitudinal and interventional studies are required to disentangle cause from consequence and to determine whether modulation of the microbiome can indeed modify sex-dependent cardiovascular risk or inform future sex specific diagnostic approaches and therapeutic options.

## Supplementary Information

Additional file 1.

## Data Availability

All data generated or analysed during this study are included in this published article and its supplementary information files.

## Abbreviations

ACSS2: Acyl-CoA synthetase short-chain family member 2
ALK5: Activin receptor-like kinase 5
AMI: Acute myocardial infarction
BA: Bile acids
BCAA: Branched-chain amino acids
BMI: Body mass index
CA: Cholic acid
CDCA: Chenodeoxycholic acid
CAD: Coronary artery disease
CVD: Cardiovascular disease
CYP7A1: Cytochrome P450 family 7 subfamily A member 1
CYP8B1: Cytochrome P450 family 8 subfamily B polypeptide 1
DCA: Deoxycholic acid
EndMT: Endothelial-to-mesenchymal transition
FMO: Flavin-containing monooxygenase
FXR: Farnesoid X receptor
HDCA: Hyodeoxycholic acid
IFN-γ: Interferon γ
IL-6: Interleukin 6
IS: Indoxyl sulfate
LCA: Lithocholic acid
LPS: Lipopolysaccharides
MACE: Major adverse cardiovascular events
MESA: Multi-Ethnic Study of Atherosclerosis
NKT: Natural killer T cells
NOX4: NADPH Oxidase 4
SCFA: Short-chain fatty acids
SMADs: Intracellular mediators of TGF-β/ALK5 signaling
STEMI: ST-segment elevation myocardial infarction
TGR5: Takeda G protein-coupled receptor 5
TMA: Trimethylamine
TMAO: Trimethylamine-N-oxide
TNF-α: Tumor Necrosis Factor α
TF: Tissue factor
