# Supplementary material for: The gut–heart axis in coronary artery disease: a scoping and narrative review of sex-based microbial and metabolic disparities
Source: Biol Sex Differ. 2026 Jan 30;17:24. doi: 10.1186/s13293-026-00824-w (PMC12884622; doi:10.1186/s13293-026-00824-w)
Supplement: Supplementary file 1 — Additional file 1. [file 13293_2026_824_MOESM1_ESM.pdf]

Appendix 1:

Limits to apply if any:

- Peer reviewed published articles only
- Conference abstracts removed

Data bases and number of results:

Database name	Dates covered	Number of records found	Final date searched
Pubmed	01.04.2015-31.03.2025	217	01.04.2025
Embase (OVID)	01.04.2015-31.03.2025	69	01.04.2025

Total number of records retrieved: 286

Total number of records after de-duplication: 254

Strategies: copy and pasted in Pubmed

("Coronary Artery Disease"[Mesh] OR "Acute Coronary Syndrome"[Mesh] OR "Myocardial Infarction"[Mesh] OR ((coronary OR ischemic OR ischaemic) AND (disease OR syndrome OR event OR infarct* OR atherosclero* OR thrombosis)) OR ("acute coronary syndrome" OR "myocardial infarction" OR STEMI OR NSTEMI) OR ("heart attack" OR "coronary stenosis" OR "coronary occlusion")) AND ("Gastrointestinal Microbiome"[Mesh] OR ((gastrointestin* OR gut OR intestin*) AND (microbiome OR microbiota OR flora OR microflora OR microbe*)) OR (bacteria AND (intest* OR gut OR enteric)) OR (Firmicutes OR Bacteroidetes OR Actinobacteria OR Proteobacteria OR Fusobacteria OR Verrucomicrobia) OR "Cholic Acid"[Mesh] OR "Deoxycholic Acid"[Mesh] OR "Glycocholic Acid"[Mesh] OR "Taurocholic Acid"[Mesh] OR "Picolinic Acids"[Mesh] OR "Serotonin"[Mesh] OR "Tryptamines"[Mesh] OR "Acetic Acid"[Mesh] OR "Butyric Acid"[Mesh] OR "Carnitine"[Mesh] OR ("Cholic Acid" OR "Deoxycholic Acid" OR "Glycocholic Acid" OR "Chenodeoxycholic Acid" OR "Glycochenodeoxycholic Acid" OR "Glycoursodeoxycholic Acid" OR "Hyodeoxycholic Acid" OR "Lithocholic Acid" OR "Taurochenodeoxycholic Acid" OR "Taurocholic Acid" OR "Taurodeoxycholic Acid" OR "Tauroursodeoxycholic Acid" OR "Taurolithocholic Acid" OR "Ursodeoxycholic Acid") OR ("Indole-3*" AND "acid") OR ("indole-3-carbinol") OR ("Skatol" OR "Tryptamine" OR "5-hydroxytryptamine" OR "3-hydroxykynurenine") OR ("Anthranilic Acid" OR "Acetic Acid" OR "Formic Acid" OR "Propanoic Acid" OR "2-methyl-propanoic Acid" OR "Butanoic Acid" OR "3-methyl-butanoic Acid" OR "Pentanoic Acid" OR "4-methyl-pentanoic Acid" OR "Hexanoic Acid" OR "Heptanoic Acid") OR ("Trimethylamine N-Oxide" OR "TMAO") OR ("Indole" OR "Carnitine" OR "Serotonin") OR ("Indoxyl" AND ("Sulfate" OR "Sulphate"))) AND ("Sex Factors"[Mesh] OR (sex difference* OR gender difference* OR "male vs female" OR "men vs women") OR (hormone* AND (estrogen OR androgen OR testosterone OR progesterone)) OR (menopaus* OR postmenopaus* OR sex-specific OR gender-specific))
